# RNA binding proteins potentially regulate alternative splicing of immune-related genes during the progression of coronary artery disease

**DOI:** 10.3389/ebm.2025.10430

**Published:** 2025-08-29

**Authors:** Yulin Miao, Lei Wang, Gang Zhao, Wei Gou, Shan Chen, Chao Ding, Zongxin Li, Fengli Gao

**Affiliations:** ^1^ Department of Vascular Surgery, General Hospital of Ningxia Medical University, Yinchuan, Ningxia, China; ^2^ The First Clinical Medical College, Ningxia Medical University, Yinchuan, Ningxia, China

**Keywords:** RNA-binding proteins, coronary artery disease, alternative splicing, co-expression, immune cells

## Abstract

RNA-binding proteins (RBPs) are crucial in disease as they regulate the biological functions of RNA. However, their role in coronary artery disease (CAD) progression remains unclear. RNA-seq from peripheral blood of CAD patients and no-CAD controls was analyzed to compare differentially expressed genes (DEGs) and explore their potential functions. The distribution of immune cells was assessed by CIBERSORT algorithm. Alternative splicing (AS) pattern was quantified by SUVA. Immune-related AS events (ASEs) were screened via ImmPort database. Co-expression network of ASEs, differentially expressed RBPs (DERBPs), mitochondrion and apoptosis genes, and immune cells was constructed to clarify their potential functions. A total of 1521 DEGs were detected, including 99 DERBPs, which were mainly downregulated and enriched in mRNA processing, RNA splicing, mRNA transport, and innate immune response pathways in CAD. Seven DERBPs (ANG, C4BPA, DDX60, IFIH1, IPO7, MATR3, OTUD4) were associated with immune function. Analysis of the immune cell fraction demonstrated significant increase in macrophage M0 and CD8 T cells and decrease in resting dendritic cells and activated memory CD4 T cells. Immune-related ASEs correlated with atherosclerotic stenosis were mainly the complex “alt3p/alt5p” splicing types. DERBP-AS’s co-expression identified a key A5′SS event of CTSB gene. Co-expression of this event with TST and SYNCRIP may lead to a change in the proportion of macrophage M0 and CD8 T cells, respectively. The mitochondrion and apoptosis genes were also dysregulated in CAD and correlated with four DERBPs. In conclusion, RBPs have potential regulatory role in the progression of CAD by regulating the ASEs of immune-related genes and mediating immune cells composition. These findings highlight RBPs as potential therapeutic targets for CAD.

## Impact statement

RNA-binding proteins (RBPs) play important functions in diseases because they regulate the biological function of RNA. However, their role in the pathogenesis of coronary artery disease remains unclear. By analyzing peripheral blood RNA sequencing data of CAD patients and healthy controls, this study found dysregulated RBPs expression in CAD patients, and constructed a potential regulatory network of dysregulated RBPs, immune cells and alternative splicing events. Further analysis suggests that RBPs may influence immune microenvironment remodeling by modulating the alternative splicing pattern of immune genes, thereby promoting the progression of CAD. This study illustrates the important function of RBPs in the pathogenesis of CAD and provides potential targets for CAD treatment in future.

## Introduction

Coronary artery disease (CAD) is a group of cardiovascular diseases (CVDs) that lead to myocardial ischemia, hypoxia, necrosis and dysfunction due to insufficient blood supply or blockage of coronary arteries [1]. Despite the continuous development of medicine, CAD is widely diagnosed worldwide, and is recognized as one of the primary causes of mortality and disease burden related to CVD [2]. Atherosclerosis is a major cause of CAD, and atherosclerotic stenosis is an inflammatory phenomenon caused by the accumulation of lipid particles and enrichment of inflammatory cytokines, resulting in structural abnormalities in the vessel lumen [3]. In patients with CAD, lipid plaques form in the lining of the coronary arteries, leading to narrowing of the vessel lumen, preventing normal blood supply to the heart muscle. Over time, lipid plaques gradually increase in size and may rupture, forming thrombi that further block the coronary arteries and cause acute ischemia [4]. As bioinformatics technology develops, studies continue to uncover the underlying mechanisms that mediate the development of CAD, which may lead to new approaches to treating CAD. Immune responses mediate homeostasis and damage repair in cardiac physiology and contribute to metabolism and tissue clearance in the healthy heart. However, long-term immune responses that are not controlled can result in adverse cardiac remodeling and further deterioration of cardiac function [5]. In CAD, each immune cell response may play a crucial role. However, the molecular mechanisms of immune cells involved in developing CAD are not yet fully understood and the exact causes remain unclear.

It has been reported that innate immune cells (such as neutrophils, monocytes, and macrophages) and adaptive immune cells (such as T and B cells) interact closely in the pathogenesis and development of atherosclerosis [3]. Macrophages and lymphocytes exist at all stages of plaque formation and promote CAD progression. A significant increase in inflammatory macrophages can lead to a larger area of atherosclerotic plaque [6]. In CAD patients, the CD8^+^ T lymphocytes in the peripheral blood is significantly increased, leading to increased production of interferon γ, which exacerbates the atherosclerotic process [7]. In addition, immune cells can accelerate the development of atherosclerosis by regulating the production and circulation of monocytes [8]. Meanwhile, it is not determined how the immune cells are regulated during the development of CAD.

RNA binding proteins (RBPs) can regulate transcription, splicing, modification, translation, localization and transport by binding to target RNAs [9]. RBPs mediate RNA regulation and control translation, which are crucial in angiogenesis [10]. RBPs have critical functions in the initiation, progression and rupture of atherosclerotic plaques by regulating endothelial and vascular structure, immune cell infiltration, and lipid accumulation [11]. Alternative splicing (AS) is one molecular process by which precursor mRNAs (pre-mRNAs) of the same gene can be spliced differently to produce multiple mature mRNAs [12]. Abnormal alternative splicing can result in protein dysfunction, which can lead to various diseases [13]. RBPs are currently a major focus in the study to explore the molecular mechanisms of CAD, and the AS of RBPs and pre-mRNAs has emerged as a key regulator of CAD and is considered to be potential therapeutic targets. The differential expression of RBPs not only affects the tissue-specific splicing pattern, but also affects the alternative splicing transcript of CAD prognostic risk [14]. Further in-depth research is required to investigate how RBPs regulate AS in CAD progression, as there are limited studies on this topic.

In this study, we analyzed transcriptome sequencing data (RNA-seq) from peripheral blood of patients with high coronary artery stenosis (CAD) and healthy controls without coronary artery disease (no-CAD), and focused our attention on the dysregulated RBPs and their potential regulatory functions on the immune cell fractions and alternative splicing pattern within these two groups. We found that the expression of RBPs is dysregulated in the CAD group and may influence the remodeling of the immune microenvironment by regulating alternative splicing of immune-related genes, which expands our understanding of the pathogenesis of CAD.

## Materials and methods

### Retrieval and process of public data

The published RNA-seq dataset (GSE202625) was downloaded from the Sequence Read Archive (SRA) database with SRA run files, which were then converted to fastq files by NCBI SRA Tool fastq-dump (v.2.8.0). The low-quality bases of raw sequencing reads were trimmed using the FASTX-Toolkit (v.0.0.13;^1^). The removal criteria were the base with terminal mass less than 20 and 30% of the reads whose base quality was less than 20. Then we used to the FastQC^2^ check the quality of filtered reads.

### RNA-seq alignment and differentially expressed gene (DEG) analysis

The quality-filtered reads were then aligned to the human GRCh38 genome via HISAT2 software (v.2.2.1) [15]. Uniquely aligned reads were used to calculate the reads count located on each gene. The normalized expression level of each gene was evaluated using fragments per kilobase of exon per million fragments (FPKM). The DESeq2 (v.1.30.1) software [16], which analyzes the differential expression between samples, was used to perform differential gene expression analysis using the reads count file, and obtain the DEGs with the fold change (FC ≥2 or ≤0.5) and false discovery rate (FDR ≤0.05).

### Extraction of differentially expressed RBPs

Then we extracted the differentially expressed RBPs from all the DEG set according a catalog of 2,141 RBPs that were retrieved from three previous reports [17–19].

### Alternative splicing identification and dysregulation analysis

According to the alignment of RNA-seq dataset, the alternative splicing events (ASEs) were identified and quantified by splicing ratios using the SUVA (v2.0) software [20]. The different splicing pattern of each group was analyzed. Reads proportion of SUVA AS event (pSAR) of each AS events were calculated to identify the differential ASEs.

### Analysis on immune-related genes

To analyzed the expression pattern of immune-related genes, we retrieved 1793 immune-related genes from the ImmPort database^3^ to identify their expression pattern and make an overlap analysis with DEGs in this study.

### Analysis on mitochondria-related genes

We also analyzed the expression pattern of 1136 mitochondria-related genes that were extracted from the Human MitoCarta3.0 database [21].

### Analysis on apoptosis-related genes

The 87 apoptosis-related genes were obtained from Molecular Signatures database (GSEA | MSigDB).

### Cell-type quantification

We used the CIBERSORT algorithm (v1.03) [22] to estimate immune cell fractions with the default parameter. The FPKM values of each expressed gene were used as input. We identified the fractions of 22 human immune cell phenotypes in the study, which have been detailed described in the results part. The fraction difference of each immune cell type were also calculated between CAD and normal groups.

### Co-expression analysis

The co-expression analysis of RBP and immune cells was constructed by calculating the Pearson’s correlation coefficient between RBP and immune cells. We finally screened out the RBP and immune cell correlation pairs with absolute correlation coefficient ≥0.6 and *P*-value ≤0.01.

The co-expression analysis was performed for immune-related ASEs and RBPs co-expressed with immune cells. Meanwhile, Pearson’s correlation coefficient among them was calculated. Immune-related ASE and RBP co-expressed with immune cells pairs with absolute correlation coefficient ≥0.6 and *P*-value ≤0.01 were screened.

### Functional enrichment analysis

The enriched Gene Ontology (GO) pathways for selected gene sets were identified using KOBAS 2.0 [23] by calculating p-value using Hypergeometric test and Benjamini-Hochberg FDR controlling procedure to define the significance of each pathway.

### Statistical analysis

Principal component analysis (PCA) for expressed genes was performed by R package factoextra^4^ to show the clustering of CAD and non-CAD samples with the first two components. The in house-script sogen was used to visualize the genomic distribution and structure of aligned reads for selected genes by normalizing their expression using TPM (Tags Per Million). The pheatmap package^5^ in R was used to perform gene expression clustering based on Euclidean distance. Student’s *t*-test was used for statistical comparison between two groups. The Venn diagram for overlapping analysis is produced using jvenn website (Venn Diagrams-jvenn) [24].

## Results

### RNA-seq analysis of peripheral blood cells from patients with CAD and no-CAD donors revealed DERBPs

**FIGURE 1:**
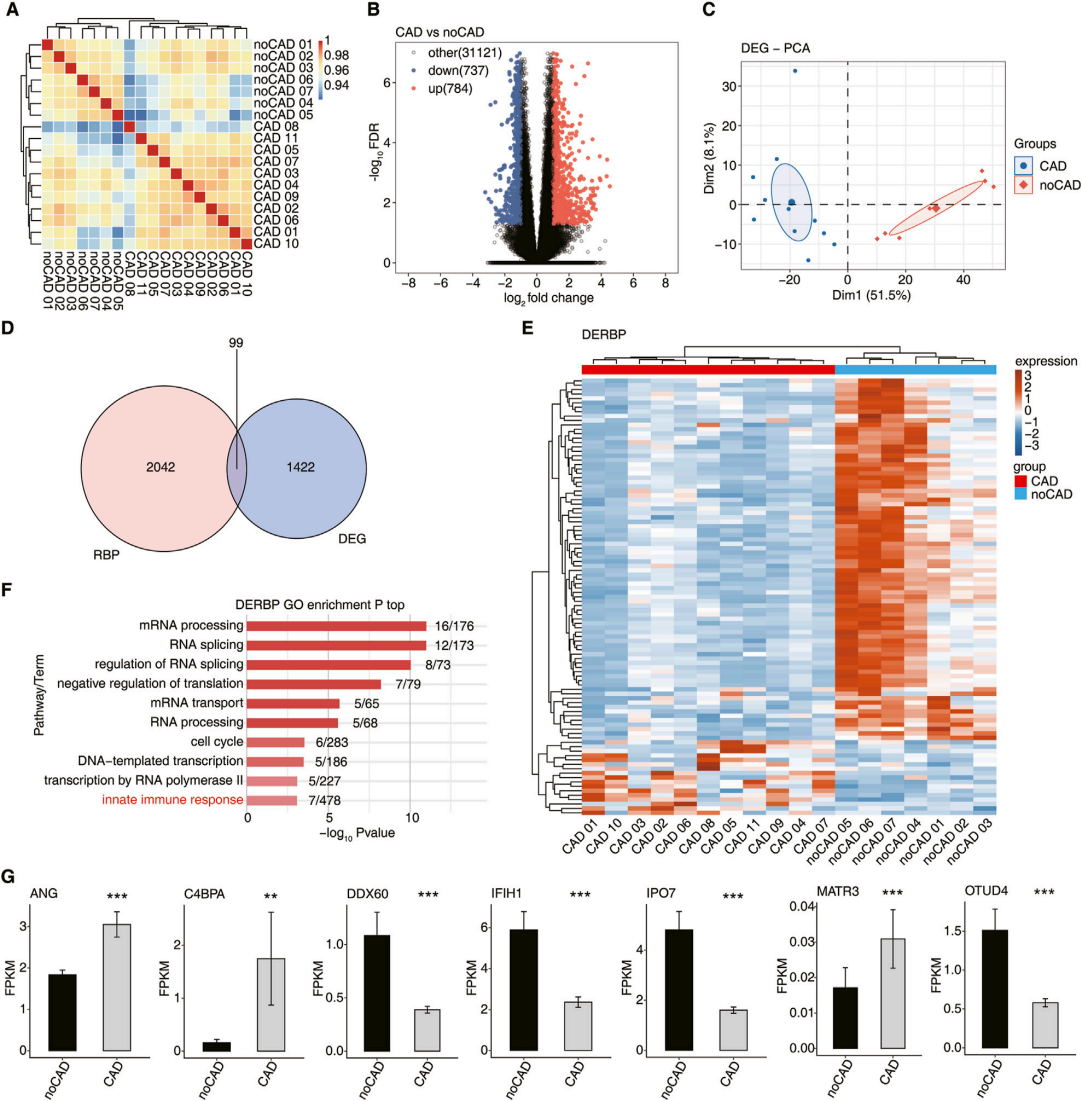
Expression profile analysis of differentially expressed RBPs in coronary artery disease. **(A)** Hierarchical clustering heatmap showing correlation between CAD and no-CAD samples based on the normalized values of all genes. **(B)** Volcano plots presenting differential pattern of all DEGs between CAD and no-CAD samples. **(C)** PCA result based on FPKM value of all DEGs. The ellipse represents for the confidence ellipse. **(D)** Venn diagram showing overlapped genes of DEGs and RBPs. **(E)** The heatmap diagram showing the expression profile of DERBP. **(F)** Bar plot showing the top enriched GO biological process results of DERBP. **(G)** The bar plot showing the FPKM values of 7 DERBPs. *: *P*-value ≤0.05, **: *P*-value ≤0.01, ***: *P*-value ≤0.001.

The RNA-seq data for GSE202625 were downloaded and re-analyzed from the GEO database and consisted of 27 CAD patients and 25 no-CAD controls. All CAD patients had confirmed severe coronary atherosclerosis (>70% stenosis in at least one vessel) by coronary computed tomography angiography (CCTA), whereas no-CAD controls had no atherosclerosis. Samples with correlation coefficients ≥0.9 or higher were first extracted, and then outlier samples were excluded; 11 CAD patients and 7 no-CAD controls were finally included in our study. First, we calculated the correlation coefficients for all the samples to evaluate the expression similarity of all samples and their comparability (Figure 1A). The results indicated that the samples within the group were highly correlated and suitable for further analyses. Then, we identified 1521 differentially expressed genes (DEGs) between CAD vs. no-CAD groups. Of these, 784 genes were upregulated and 737 genes were downregulated in the CAD group (Figure 1B). The GO analysis of up-regulated DEGs demonstrated that they were significantly enriched in pathways related to immune response, while the down-regulated DEGs were mainly enriched in cell division, cell cycle, DNA repair, and mRNA processing pathways (Supplementary Figures S1A,B). To validate the classification results based on DEGs, PCA was performed in this study. The two groups were clearly separated by the first or top component (Figure 1C), suggesting that the DEGs are of research significance.

Among the detected DEGs, we found 99 DERBPs (Figure 1D). The expression distribution of DERBPs showed obvious difference between the CAD and no-CAD groups, and most of these DERBPs were down-regulated in CAD (Figure 1E). GO analysis demonstrated that these DERBPs were enriched in biological pathways including mRNA processing, RNA splicing, mRNA transport and innate immune response (Figure 1F). DERBPs are primarily associated with RNA post-transcriptional regulation, and some are also involved in immune response biology. We selected seven immune-related DERBPs, including *ANG, C4BPA, DDX60, IFIH1, IPO7, MATR3*, and *OTUD4*, and found they were significantly differentially expressed between CAD vs. no-CAD groups (Figure 1G). To sum up, there results demonstrate that DERBPs may influence CAD development by mediating immune responses and their potential downstream target genes.

### Dynamic changes of DERBP were associated with immune microenvironment regulation in coronary artery disease group

**FIGURE 2:**
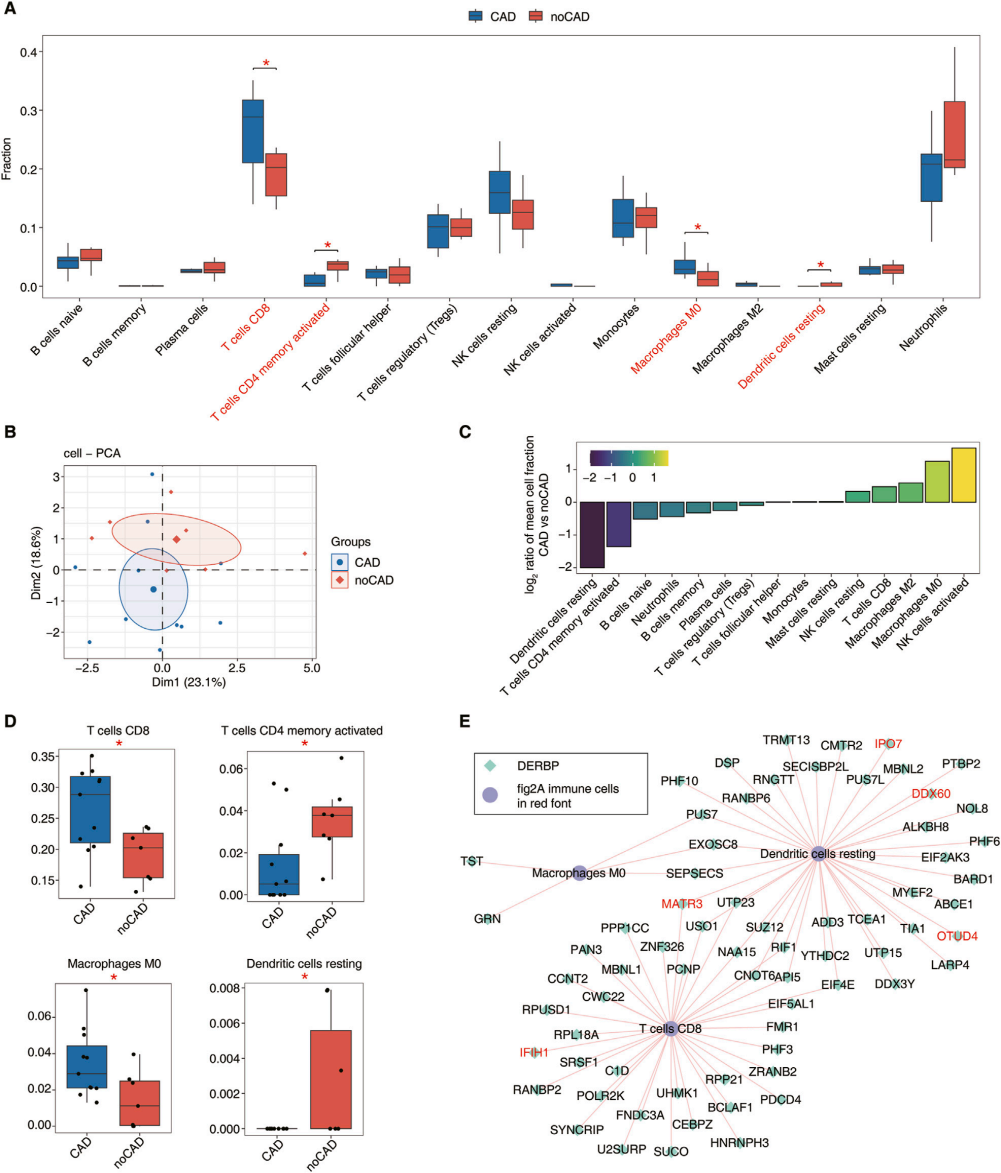
Dynamic changes of DERBP related to immune microenvironment regulation in coronary artery disease samples. **(A)** Boxplot showing percentage of immune cell types in CAD and no-CAG groups. The statistical difference between CAD and no-CAD samples was calculated by the Student’s *t*-test. *: *P*-value ≤0.05, **: *P*-value ≤0.01, ***: *P*-value ≤0.001. **(B)** PCA result based on percentage of immune cell types. The ellipse for each group is the confidence ellipse. **(C)** Bar plot showing the relative frequency ratio at CAD vs. no-CAD based on decreasing values of ratio. **(D)** Boxplot showing the percentage of 4 significant immune cells. **(E)** Co-expression analysis of DERBP and Panle **A** immune cells in red font. The network showing the Co-expression of DERBP and immune cells in red font.

To further investigate the composition differences of immune cells in the CAD patients, we used CIBERSORT immune cell infiltration method to analyze the scores of different immune cells between CAD vs. no-CAD samples for the 15 immune cell types (Supplementary Figure S2A). Immune cell type analysis was performed on samples from both groups, and we found the significant difference (*P* < 0.05) for the proportions of macrophage M0, CD8 T cells, resting dendritic cells, and activated memory CD4 T cells between the CAD and no-CAD groups (Figure 2A). The PCA plot showed the variability in the percentage of immune cells between the two groups, which is sufficient to show that there is substantial variability in the proportion of immune cells in the peripheral blood of CAD patients (Figure 2B). Based on the ratio of the relative proportions of different immune cells, we found an increase in macrophage M0 and CD8 T cells and a decrease in resting dendritic cells and activated memory CD4 T cells in the CAD group (Figure 2C). The proportion difference of CD8 T cells, CD4 T cells, macrophage M0 and resting dendritic cells was significant (*P* < 0.05), and we further demonstrated the distribution of these four immune cell types between the two groups (Figure 2D). The changes in immune cells indirectly suggest that the development of CAD may be related to the immune cell microenvironment.

To investigate how DERBPs associated with the immune cell microenvironment in CAD, we performed co-expression analysis between immune cells and DERBPs. It was found that 36 DERBPs were associated with CD8 T cells, macrophage M0 was correlated with 5 DERBPs, and resting dendritic cells were correlated with 39 DERBPs (|correlation| ≥0.6, *p* ≤ 0.01) (Figure 2E). In addition, neutrophils, out B cells and activated NK cells were similarly co-expressed with a small number of DERBPs (Supplementary Figure S2B). We found that some of these DERBPs co-expressed with immune cells are directly related to the innate immune response, such as MATR3 and IFIH1 co-expressed with CD8 T cells, and IPO7, DDX60, OTUD4 and MATR3 co-expressed with the resting dendritic cells. From the above co-expression relationships, we demonstrate that the expression changes of DERBPs can modulate the proportion of immune cells, and that a series of changes may contribute to the development of CAD.

### Co-disturbed regulatory network of immune-related RBPs and ASE was constructed in CAD

We have identified the 99 DERBPs existed between the CAD and no-CAD groups, and the expression of some DERBPs showed significant correlation with the proportion of immune cells, but how DERBPs affect peripheral blood immune cell changes in CAD patients is still unclear. RBPs are known to regulate pre-mRNA alternative splicing. Next, we focus on the co-expression relationship among DERBP, immune cells, and differentially expressed immune-related ASEs, to identify the alternative splicing of immune genes that may be regulated by DERBPs and further clarify the potential regulatory relationship between RBPs and immune-related ASEs in CAD.

**FIGURE 3:**
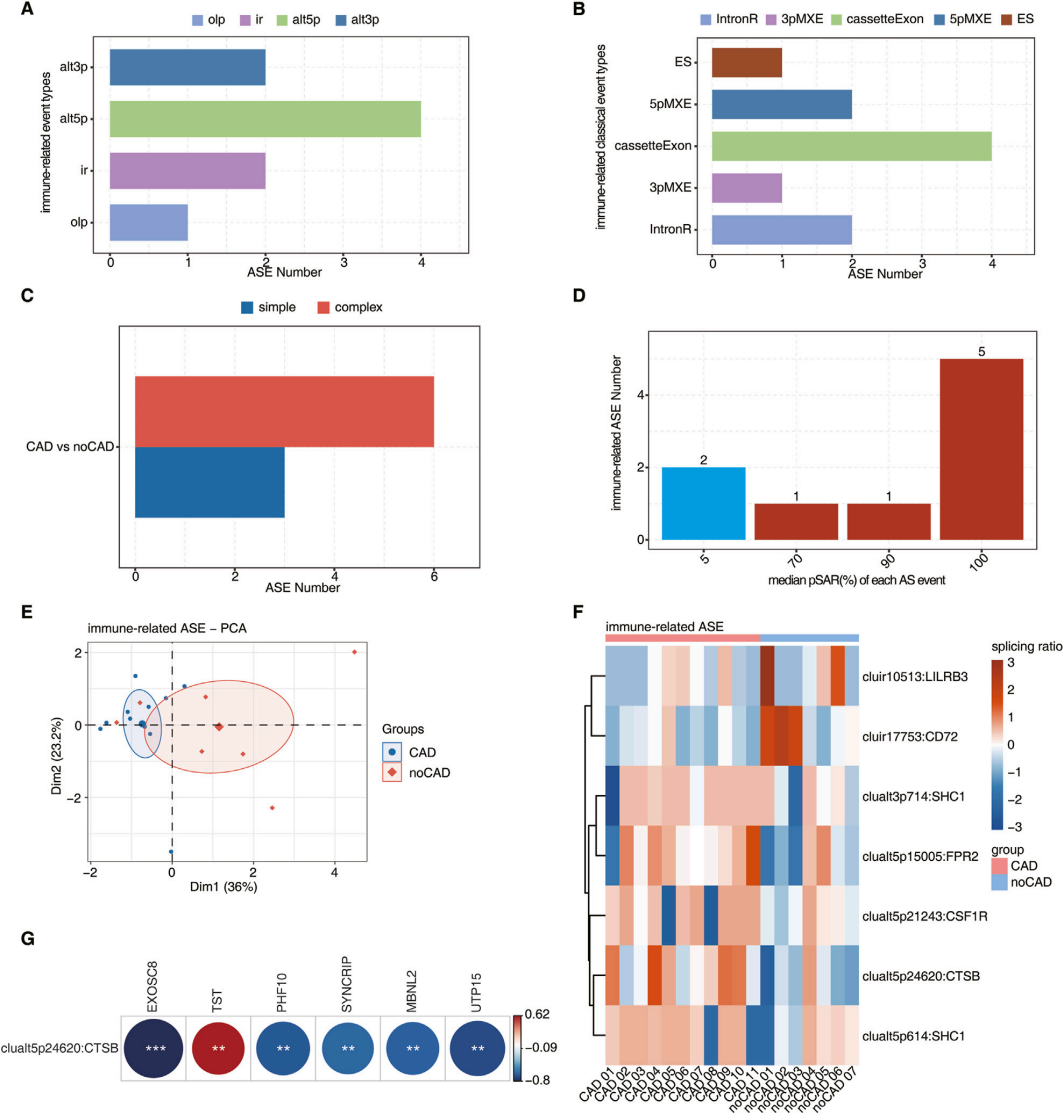
Analysis of immune-related SUVA in coronary artery disease and its co-expression of DERBP associated with immune microenvironment regulation. **(A)** Bar plot showing the number of immune-related regulatory AS detected by SUVA. **(B)** Splice junction constituting immune-related AS events detected by SUVA was annotated to classical AS event types. And the number of each classical AS event types were showed with bar plot. **(C)** Bar plot showing the number of simple and complex splicing events in immune-related AS events. **(D)** Bar plot showing immune-related ASE with different pSAR. ASE which pSAR (Reads proportion of SUVA AS event) ≥50% were labeled. **(E)** Principal component analysis (PCA) based on immune-related ASE of pSAR ≥50%. The ellipse for each group is the confidence ellipse. **(F)** The Heatmap showing the splicing ratio of immune-related AS events (PSAR ≥50%) in the CAD vs. no-CAD group. **(G)** The heatmap diagram showing co-expression analysis of immune-related AS events, RBP co-expressed with immune cells. Cutoffs of p value ≤0.01 and Pearson coefficient ≥0.6 or ≤−0.6 were applied to identify the co-expression pairs.

We first analyzed all ASEs (Supplementary Figures S3A–F) that occurred in the CAD and no-CAD groups using the SUVA method, and then screened for immune gene-related ASEs by ImmPort database. The major differential immune-related ASEs identified by SUVA were ‘alt3p/alt5p’ event types (Figure 3A). The immune-related ASEs identified by SUVA were then matched with classical splicing events, which were mainly cassetteExon and 5pMXE types (Figure 3B). Meanwhile, the immune-associated ASEs in CAD were predominantly complex splicing events (Figure 3C), illustrating the complexity of CAD-related splicing in modulating the immune response.

One ASE contains at least two RNA isoforms, and one isoform may only account for a small part of the total RNA molecules of this gene, so we would like to find the dominant transcript. The splicing rate pSAR (proportion of SUVA AS event Reads) was used to calculate the proportion of each isoform in which the ASE is located and compared to the total isoform levels of the entire gene; a lower pSAR indicates that the two transcripts involved in splicing account for a smaller proportion of gene expression. Figure 3D showed the number distribution of ASEs that account for different proportions of all reads in the region, with a significant proportion of ASEs accounting for only a small proportion. We selected 7 immune-related ASEs that were the predominant transcripts (pSAR >= 50%) for subsequent analyses, and a small number of immune-related ASEs played a major role in CAD disease by performing PCA using their splicing ratios (Figure 3E). The ASEs of key transcripts in immune-related ASEs were shown by heatmap, showing a significant difference in the splicing ratio of immune-related ASEs between CAD and no-CAD groups (Figure 3F).

To identify immune-related ASEs that may be regulated by DERBPs in CAD patients, we performed co-expression analyses between immune-related ASEs and immune cell-associated DERBPs and found that the A5′SS event of the CTSB gene (clualt5p24620:CTSB) was co-expressed with 6 DERBPs that were worthy of attention (|correlation| ≥0.6, p ≤ 0.01) (Figure 3G). Where the distribution of reads of splicing events on CTSB showed that the splicing ratio was significantly higher in CAD (Supplementary Figure S3G), suggesting that this ASE may be important for CAD formation. Considering that these 6 DERBPs were also co-expressed with immune cells, in particular TST and SYNCRIP co-expressed with macrophage M0 and CD8 T cells, respectively (Figure 2E). Therefore, we hypothesized that the TST-CTSB and SYNCRIP-CTSB co-expression pairs may affect the peripheral blood macrophage M0 and CD8 T cell ratios in CAD patients, respectively.

### DERBPs potentially regulate expression of mitochondrial and apoptotic genes

**FIGURE 4:**
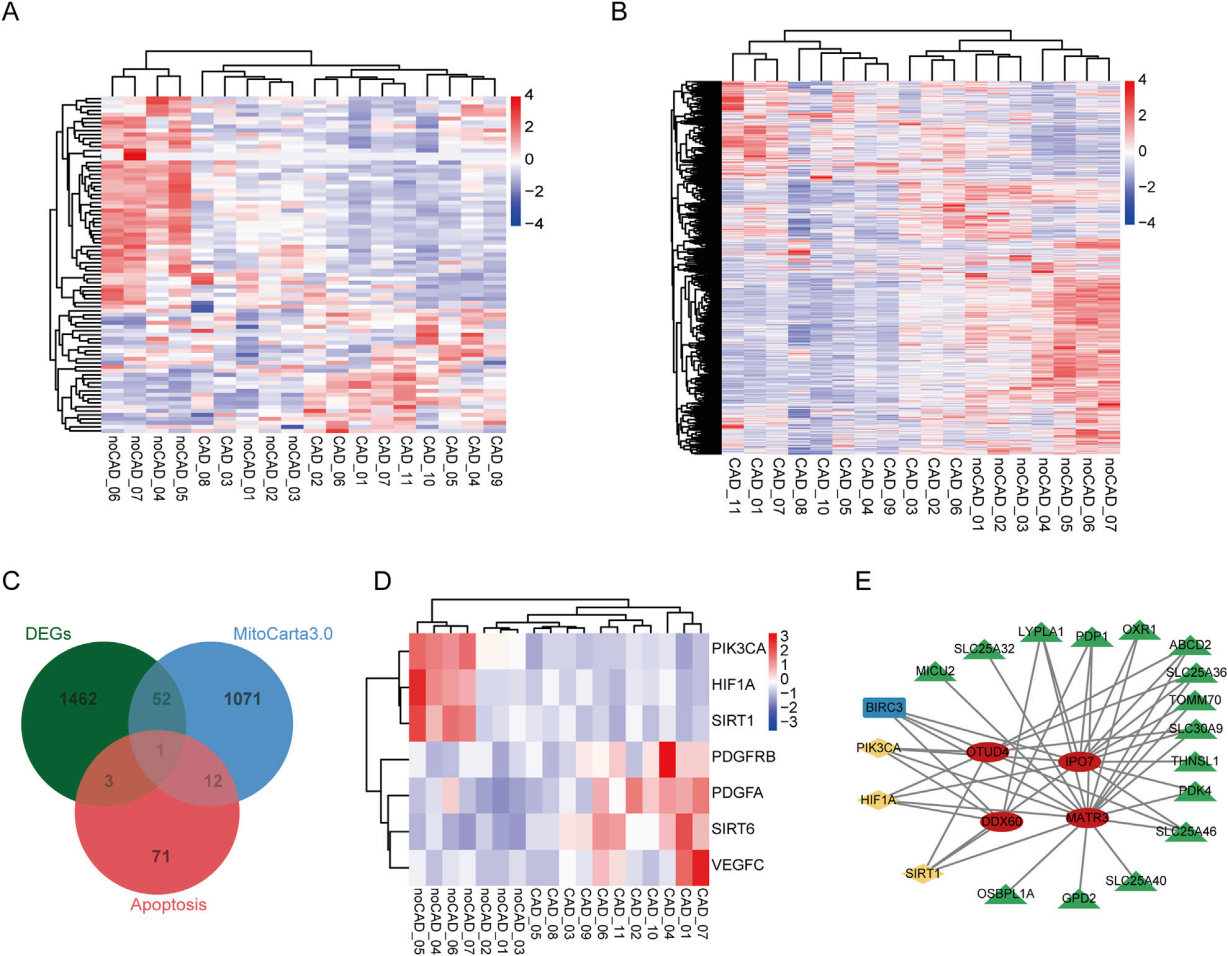
Analysis of mitochondrial dysfunction in CAD and its co-expression of seven immune-related DERBPs. **(A)** The heatmap of all apoptosis-related genes in the CAD vs. no-CAD group. **(B)** The heatmap of all mitochondria-related genes in the CAD vs. no-CAD group. **(C)** Venn diagram showing the overlap of DEGs, apoptosis- and mitochondria-related genes. **(D)** The heatmap of HIF-1α signaling related genes in the CAD vs. no-CAD group. **(E)** The mitochondrial dysfunction regulatory network of immune-related DERBPs. Red ovals represent immune-related DERBPs, green triangles represent mitochondrial-related genes, blue rectangle represents apoptosis-related genes, yellow rhombuses represent HIF-1α signaling related genes.

Mitochondria are important regulatory centers of apoptosis, for mitochondrial dysfunction activates the apoptotic signaling pathway and promotes apoptosis in various cell types. Next, we explored the expression pattern of all apoptosis-related genes and mitochondrial-related genes in CAD and noCAD patients, and found they showed obvious difference between CAD and noCAD samples (Figures 4A,B). The overlap analysis results demonstrated that there were 4 apoptosis-related genes, and 53 mitochondrial-related genes were DEGs (Figure 4C). As an important regulatory factor in cardiovascular diseases, we also analyzed the expression of HIF-1α signaling pathway, and found HIF1A was downregulated in CAD samples (Figure 4D). Although both SIRT1 and SIRT6 are inhibitors of HIF-1α, their expression changes are opposite (Figure 4D), which implies the complex role of HIF-1α signaling in CAD. To clarify whether the function of immune-related DERBPs in CAD is associated with mitochondrial dysfunction, we constructed a co-expression regulatory network by analyzing the correlations between the seven immune-related DERBPs and these DEGs. The results indicated that HIF-1α signaling might mediate the regulation of mitochondrial-related genes by OTUD4, IPO7, DDX60, and MATR3 (Figure 4E). What’s more interesting, all these correlated genes were downregulated in CAD.

## Discussion

With changing lifestyles, the incidence of CAD with high mortality is increasing every year and has become one of the most serious public health problems. Atherosclerosis is an inflammation-associated disease with immune properties, driven by an innate immune response via monocytes and macrophages, with the resulting chronic inflammatory response attracting various immune cells, including innate and adaptive immune systems, to the atherosclerotic plaques [25, 26]. In this study, we performed bioinformatics analysis to focus on characterizing the immune microenvironment in CAD by analyzing RNA-seq data from GSE202625. The potential regulatory relationship between RBPs and immune-related alternative splicing during CAD progression was explored by analyzing DEGs and differential ASEs occurring in peripheral blood samples from CAD and no-CAD patients to shed light on possible pathogenic mechanisms in CAD. Through comprehensive exploration of this dataset, we found that a large number of genes were altered between the CAD vs. no-CAD groups, and further analysis revealed 99 DERBPs. GO analysis suggested that some DERBPs were associated with immune response effects in CAD, consistent with the conclusion of a previous report on the involvement of RBPs in immune cell regulation of atherosclerotic plaques [11]. Notably, we identified seven immune related DERBPs (*ANG*, *C4BPA*, *DDX60*, *IFIH1*, *IPO7*, *MATR3*, *OTUD4*) in the innate immune response and HIF1-α pathways that may be involved in the pathogenic process of CAD by modulating the immune cell changes.

The proteins encoded by ANG are potent mediators of neovascularization and are therefore often referred to as angiopoietins [27]. Members of the ANG family all play important roles in atherosclerosis, with ANG II thought to have pro-atherogenic effects [28]. Atherosclerotic plaque rupture can induce the acute coronary syndromes, and ANG II induces macrophage apoptosis by inducing the expression of monocyte chemotactic proteins, ultimately leading to the formation of vulnerable atherosclerotic plaques [29]. ANG II type 1 receptor blockade was found to be associated with a reduction in carotid atherosclerosis by studying the effects of valsartan on carotid artery atherosclerosis [30]. ANG II plays an important role in cardiovascular disease and can be used as therapeutic targets in cardiovascular pathologies [31, 32]. C4BPA (Complement C4b-binding protein alpha chain) is a protein associated with the immune system that has a regulatory role in the complement system and plays an important role in the immune response [33]. The role and mechanism of C4BPA in atherosclerosis have not been elucidated, but one study has shown that C4BPA expression is a promising biomarker to predict clopidogrel resistance during the treatment of atherosclerosis [34, 35], and clopidogrel is mainly used to prevent and treat diseases related to atherosclerosis and thrombosis. In addition, C4BPA can affect the activity of the complement system [36, 37], which may influence the pathogenesis and development of atherosclerosis by modulating the activity of the complement system and the inflammatory response. MATR3 (Matrin 3) is a nuclear RNA/DNA-binding protein and extensively regulates gene expression pattern, which is involved in cardiac development and exists in endothelial cells and arterial smooth muscle cells of cardiac arteries and veins [38]. While endothelial cell and smooth muscle cell dysfunction have essential function in the occurrence and development of atherosclerosis, indicating the potential regulatory role of MATR3 in atherosclerosis development.

IFIH1 encodes a protein known as MDA5 (melanoma differentiation-associated protein 5) and is an important immune-related gene involved in the process of virus infections [39]. IFIH1 was found to be an immune-related hub gene of atherosclerosis and may be a potential target for patients with atherosclerosis [40]. DDX60 is a member of the DEAD-box family and was originally thought to be an antiviral protein involved in the regulation of cellular immune responses to viral infection [41, 42]. DDX60 has antiviral effects, and certain viral infections are also considered a risk factor for atherosclerosis [43]. IPO7 (Importin 7) is an important nuclear transporter protein gene that encodes a protein involved in the transport of substances within the nucleus and between the cytoplasm of cells, and plays an important role in many biological processes [44, 45]. OTUD4 (OUT deubiquitinase 4) is a protein with deubiquitinase activity that is involved in regulating the deubiquitination modification of intracellular proteins, thereby affecting protein stability and function [46, 47]. The immune response plays a key role in the pathogenesis of atherosclerosis [48]. All of these immune-related RBP genes that we have identified can potentially regulate the immune system and are directly or indirectly involved in the immune response that ultimately affects the progression of atherosclerosis. Taken together, these DERBPs are enriched in the innate immune response pathway, and may have important functions in CAD disease by modulating immune responses in different ways, which needs to be further validated in future studies.

By analyzing the distribution of immune cells, we found a significant increase in M0 macrophage and CD8 T cells and a significant decrease in activated memory CD4 T cells and resting dendritic cells in the CAD group. Current research suggests that CD8 T cells are emerging as a key cell population in atherosclerosis [49]. Studies have shown the increased proportion of CD8 T cells in the cellular distribution of atherosclerotic plaques in mice and humans [50, 51]. M0 macrophages have not been activated or polarized, and studies have shown a significant increase in M0 macrophages in CAD patients [52]. CD4 T cells can differentiate into different TH or Treg cell subtypes, which can modulate the response of other immune cells and exert direct pro- or anti-inflammatory effects, making the diverse roles of CD4 T cells in atherosclerosis. Among these cell subtypes, Treg cells have anti-atherosclerotic effects, and depletion of Treg cells accelerates atherosclerosis [53]. In addition, the distribution of resting dendritic cells was significantly downregulated in patients with myocardial infarction [54]. These studies further suggest that the distribution of immune cells correlates with the progression of CAD. Co-expression analysis of immune cells and DERBPs was performed and found that CD8 T, macrophage M0 and resting dendritic cells all correlated with some DERBPs. It suggests that the DERBPs can cause changes in the proportion of immune cells in the peripheral blood of CAD patients, indirectly suggesting that the development of CAD may be related to the microenvironment of immune cells. We also realized that our results were based on peripheral blood samples, which have been used in exploring the gene expression profile during infection with bacterial meningitis [55]. This approach can globally detect gene expression of immune cells in peripheral blood, but further studies focusing on plaque samples are also necessary to identify the infiltrated immune cells during CAD progression.

We then constructed a co-expression network of immune-related ASEs and immune-cell-related DERBPs and finally identified that the A5′SS event of the CTSB gene correlates with six DERBPs (MBNL2, UTP15, PHF10, TST, EXOSC8, and SYNCRIP) in CAD patients. CTSB (Cathepsin B) belongs to the lysosomal cathepsin family of proteases and regulates the quantity of lysosomes and autophagosomes in cells [56, 57]. CTSB has been involved in the progression of apoptosis, autophagy, and certain types of cancer [58, 59]. CTSB is also involved in the development of several cardiovascular diseases, such as atherosclerosis, heart failure, myocardial infarction and so on [60]. Additionally, it has been found that genetic variation in the CTSB promoter may affect the development of dilated cardiomyopathy [61]. These studies have shown that CTSB plays a crucial role in regulating cardiac remodeling. Therefore, CTSB is also considered a potential therapeutic target for heart failure. CTSB is involved in extracellular matrix decomposition processes, such as fibronectin, elastin, and collagen IV, which can lead to the destruction of collagen fibers in plaques and decreased plaque stability [62]. CTSB appears to play a role in autophagy at later stages of carotid atherosclerotic plaque compared to early stages [63]. Therefore, CTSB is a potential therapeutic target for atherosclerosis and is expected to provide new ideas and methods for the treatment of this disease. Based on the result that TST and SYNCRIP were co-expressed with macrophage M0 and CD8 T cells, respectively, we hypothesize that the TST-CTSB splicing event co-expression pair may affect changes in the peripheral blood macrophage M0 cell ratio in CAD patients, and that the SYNCRIP-CTSB splicing event co-expression pair may affect changes in the peripheral blood CD8 T-cell ratio in CAD patients, which may potentially exacerbate the occurrence of CAD disease.

## Conclusion

In this study, our analysis of RNA-seq data from peripheral blood of CAD patients and no-CAD controls revealed the dysregulation of RBPs expression in CAD patients, and constructed the potential regulatory network between dysregulated RBPs and immune cells, as well as the ASEs. Further analyses suggest that RBPs may contribute to the progression of CAD by influencing the remodeling of the immune microenvironment through the regulation of alternative splicing of immune-related genes. Our results indicate the important roles of RBPs in CAD progression, and suggest that these RBPs as well as their potential targets are targets for CAD treatment in future.

## Data Availability

The original contributions presented in the study are included in the article/Supplementary Material, further inquiries can be directed to the corresponding author.

## Footnotes

1
http://hannonlab.cshl.edu/fastx_toolkit/

2
http://www.bioinformatics.babraham.ac.uk/projects/fastqc

3
https://www.immport.org/shared/genelists

4
https://cloud.r-project.org/package=factoextra

5
https://cran.r-project.org/web/packages/pheatmap/index.html
