# Supplementary material for: RNA binding proteins potentially regulate alternative splicing of immune-related genes during the progression of coronary artery disease
Source: Exp Biol Med (Maywood). 2025 Aug 29;250:10430. doi: 10.3389/ebm.2025.10430 (PMC12425833; doi:10.3389/ebm.2025.10430)
Supplement: Supplementary file 1 [file Supplementaryfile1.docx]

**FigS1**


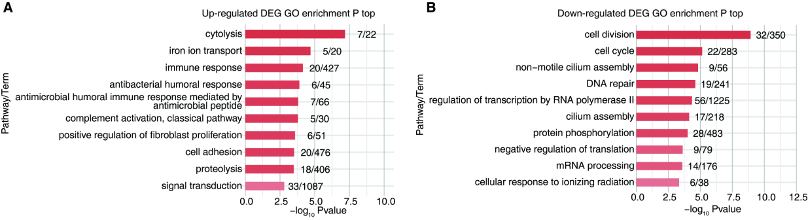


**Figure S1. Transcriptome analysis of differentially expressed RBPs in coronary artery disease.**

A. Bar plot showing the most enriched GO biological process results of the up-regulated in CAD compared with noCAD samples.

B. Bar plot showing the most enriched GO biological process results of the down-regulated in CAD compared with noCAD samples.

**FigS2**


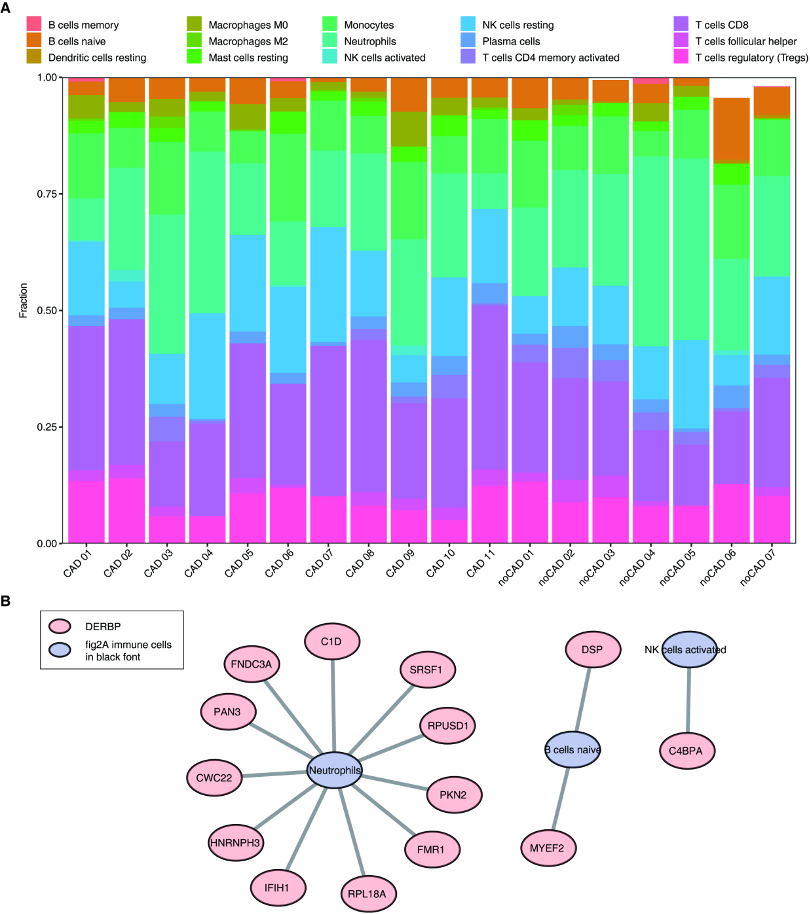


**Figure S2. Dynamic changes of DERBP related to immune microenvironment regulation in coronary artery disease samples.**

A. Fractions of different cells estimated by CIBERSORT in each sample.

B. Co-expression analysis of DERBP and fig2A immune cells in black font. Cutoffs of P value ≤ 0.01 and Pearson coefficient ≥ 0.6 or ≤ -0.6 were applied to identify the co-expression pairs. The network showing the Co-expression of DERBP and fig2A immune cells in black font.

**FigS3**


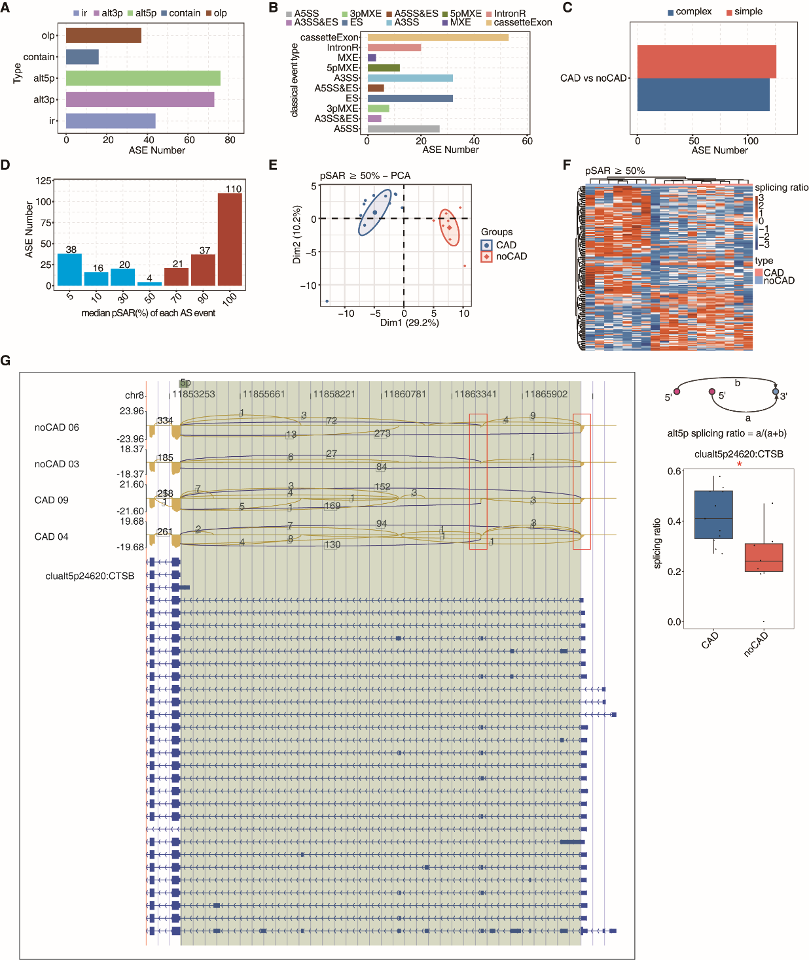


**Figure S3. Analysis of immune-related SUVA in coronary artery disease and its co-expression of DERBP associated with immune microenvironment regulation.**

1. Barplot showing number of regulatory AS detected by SUVA.
2. Splice junction constituting AS events detected by SUVA was annotated to classical AS event types. And the number of each classical AS event types were showed with barplot.
3. Barplot showing the number of simple and complex splicing events in AS events.
4. Barplot showing AS events with different pSAR. AS events which pSAR (Reads proportion of SUVA AS event) ≥ 50% were labeled.
5. Principal component analysis (PCA) based on ASE of pSAR ≥ 50%. The ellipse for each group is the confidence ellipse.
6. The Heatmap showing the splicing ratio of ASE (PSAR ≥ 50%) in the CAD vs noCAD group.
7. Reads distribution diagram showing clualt5p24620 CTSB. Boxplot showing splicing ratio of clualt5p24620 CTSB on the right. *: P value ≤ 0.05, **: P value ≤ 0.01,***: P value ≤ 0.001
